# Frequency of macroscopic intradural hemorrhage with and without subdural hemorrhage in early childhood autopsies

**DOI:** 10.1007/s12024-019-00103-8

**Published:** 2019-03-27

**Authors:** Emma C. Cheshire, Mike J. P. Biggs, Frances E. Hollingbury, Virginia L. Fitzpatrick-Swallow, Thomas R. A. Prickett, Roger D. G. Malcomson

**Affiliations:** 1 East Midlands Forensic Pathology Unit, University of Leicester, Robert Kilpatrick Building, Level 3 Leicester Royal Infirmary, Leicester, LE2 7LX UK; 2 Histopathology Department, Leicester Royal Infirmary, Infirmary Close, Leicester, LE1 5WW UK

**Keywords:** Post-mortem, Autopsy, Child, Infant, Intradural hemorrhage, Subdural hemorrhage

## Abstract

Some authors have suggested that in the fetus, neonate and infant, intradural hemorrhage (IDH) is relatively common and often presents alongside subdural hemorrhage (SDH). These authors have theorized that pediatric SDH may result from an IDH due to blood leakage from a dural vascular plexus. In this study, we report the inter-observer variation for detection of IDH from a retrospectively collected series of pediatric autopsy photographs, with and without SDH. Autopsy photographs of the falx and tentorium from 27 neonatal, infant and early childhood autopsies were assessed by two independent consultant forensic pathologists blinded to all case histories for the presence and extent (focal or diffuse) of IDH. Inter-observer agreement between the pathologists was calculated using Cohen’s kappa coefficient. The occurrence of subdural hemorrhage was also recorded at autopsy. A kappa coefficient value of 0.669 (*p* = 0.001), indicated a substantial level of agreement for the presence/absence of IDH between the pathologists. For the extent of IDH a kappa coefficient value of 0.6 (*p* = 0.038) indicated a moderate level of agreement. The pathologists agreed on the presence of IDH in 10 of the 27 cases. Subdural hemorrhage was recorded for 8 out of 27 cases. Of these 8 cases, it was agreed that 4 had IDH. Using standardized methods of image capture and assessment, inter-observer agreement for the presence/absence of IDH was substantial. In this paper, we report a much lower frequency of macroscopic IDH occurring alongside SDH than previous studies, which included both gross observation of IDH and histological examination.

## Introduction

It has been suggested that intradural hemorrhage is relatively common in the perinatal, neonatal, infant, and early childhood age groups, including in alleged cases of abusive head trauma (AHT) [1, 2]. It is particularly prevalent in the falx and tentorium [3].

Subdural hemorrhage is often seen in cases of AHT, with the most commonly suggested source of hemorrhage being traumatic damage to blood vessels, known as bridging veins, which traverse from the surface of the brain to the dural membrane. However, over the last two decades, alternative theories have emerged which suggest that infantile subdural bleeding may result from hypoxia, brain swelling and raised central venous pressure [1] and that the source of blood leakage is arterial and venous plexuses within the dura itself [3]. These theories, therefore, suggest that an intradural hemorrhage (IDH) occurs, subsequently producing a SDH via leakage out of the dura [4].

For IDH to result in SDH that is macroscopically visible at post-mortem, one might expect that the area of bleeding within the dura would need to be extensive enough to diffuse out of the membrane, into the subdural compartment, as proposed.

Our previous development of novel post-mortem techniques to remove the infant calvarial bones without damage to the dura [5] and careful dissection of the brain (including separate hemispherectomy) has allowed us to carefully document bridging veins and subdural bleeds [6]. The use of hemispherectomy and a standardized approach to photography of the brain and dura for these purposes created an archive of photos of the falx and tentorium in situ in a series of pediatric cases. Using our detailed dissection methods, we did not feel that macroscopically significant IDH was a common observation, in contrast to observations made in previous publications, particularly in the infant and young child age groups.

In this paper, we describe the occurrence of macroscopically visible intra-falcine and intra-tentorial hemorrhages, observed by two consultant forensic pathologists, in a series of photographs from 27 neonatal, infant and early childhood post-mortem examinations. We also describe several cases with documented SDH, to help elucidate whether or not the previously suggested link between IDH and SDH was evident in our study cohort.

## Materials and methods

### Case selection

Twenty seven neonatal, infant and early childhood (up to 2 years of age) autopsies undertaken at Leicester Royal Infirmary, between January 2014 and March 2016 as part of a regional pediatric autopsy service, were included in this study. Corrected gestational ages were recorded for neonates within the first 4 weeks of life (Table 1). Inclusion criteria for the series of autopsy photographs included in this study were; cases in which brain removal occurred by right hemispherectomy (enabling photography of the right falx cerebri), followed by left hemispherectomy (enabling photography of the left falx cerebri and tentorium cerebri) and cases which had 3 images of sufficient quality to demonstrate full views of both sides of the falx cerebri and a superior view of the tentorium cerebelli. Subsequently, a total of 81 post-mortem images were assessed for the presence of intradural hemorrhage by two independent consultant forensic pathologists (MJPB, FEH) blinded to all case histories. Retrospective ethical approval was obtained for the use of archived autopsy photographs of pediatric brains for research purposes (NRES 14/EM/0169).

**Table 1:** Cause of death and associated features, Pathologist A and B’s observations of macroscopic IDH from autopsy photographs and the occurrence of SDH from autopsy reports

Case #	Sex	Age (and delivery mode for neonates	Cause of death/Associated features	Macroscopic presence of IDH		SDH
		Neonates (˂28 days)		Pathologist A	Pathologist B	
1	M	36 + 3 GA, CS	HIE	Diffuse	Focal	No
2	M	1 day, NVD	Birth-related head injury	Diffuse	Diffuse	Yes
3	M	3 days, CS	HIE, uteroplacental insufficiency and ruptured vasa previa	Focal	Focal	No
4	M	3 days, CS	Persistent pulmonary hypertension of the newborn, PDA	Focal	Focal	No
5	M	3 days, CS	HIE, perinatal asphyxiation, uteroplacental insufficiency	No	No	No
6	F	3 days, FA	Pulmonary haemorrhage, subtle congenital abnormalities	Diffuse	Diffuse	Yes
7	F	8 days, NVD	Lung dysplasia	No	No	No
8	F	12 days (35 + 5 GA), FA	Positional asphyxia, co-sleeping	Focal	Focal	Yes
9	M	24 days, NVD	Pulmonary haemorrhage	Focal	Focal	No
		Infants (4 weeks - 12 months)				
10	M	4 weeks	Unascertained, SUDI, uncertain sleeping arrangement	Diffuse	Diffuse	Yes
11	M	4 weeks	Pulmonary haemorrhage	No	No	Yes
12	F	5 weeks	Positional asphyxia	Diffuse	Focal	No
13	M	8 weeks	Unascertained, SUDI, co-sleeping	Focal	No	No
14	M	9 weeks	External airway obstruction, co-sleeping	No	No	No
15	M	9 weeks	Head injury, suspected AHT (33 h survival)	Focal	Unable	Yes
16	M	10 weeks	Viral bronchiolitis	No	No	No
17	M	14 weeks	Overlaying, minor crush injury to head, co-sleeping	No	No	Yes
18	M	15 weeks	SIDS	No	No	No
19	F	16 weeks	Positional asphyxia, restrictive seating device	Focal	Focal	No
20	M	20 weeks	Unascertained, SUDI	No	Focal	No
21	F	27 weeks	Dog attack, head injury	No	No	Yes
22	F	29 weeks	Smoke inhalation	No	No	No
23	M	45 weeks	RSV bronchiolitis	No	Focal	No
		Young Children (>12 months)				
24	F	13 months	Unascertained, possible external airway obstruction	No	No	No
25	F	18 months	Unascertained, SUDI, prone sleeping, recurrent febrile convulsions	No	Focal	No
26	F	20 months	Sharp force extracranial trauma	No	Unable	No
27	M	29 months	Cystic encephalomalacia, TTTS, epilepsy	No	Unable	No

*GA* gestational age, *CS* caesarean section, *NVD* normal vaginal delivery, *FA* forceps assisted, *HIE* hypoxic ischemic encephalopathy, *PDA* patent ductus arteriosus, *SUDI* sudden unexplained death in infancy, *AHT* abusive head trauma, *SIDS* sudden infant death syndrome, *RSV* respiratory syncytial virus, *TTTS* twin to twin transfusion syndrome

### Presentation and evaluation of images

The 27 cases were randomized by pairing each case with a computer-generated decimal number between 0 and 1 using the RAND function in Microsoft Excel 2013. The randomized cases were presented in a Microsoft PowerPoint 2013 slideshow for independent assessment. The order in which the cases were shown was determined by the randomly generated decimal numbers (from the smallest to largest value). There was one image per slide, and all images were of the same dimensions (16.62cm × 24.92cm). Case images were viewed on a 23″ LED backlit monitor (HP Compaq L2306x) with a privacy screen.

The researcher responsible for the design of the test and the randomization of the images (TRAP) was also blinded to the case histories. After statistical analysis of the data, case details and causes of death were provided from the autopsy reports, by the consultant pediatric and perinatal pathologist (RDGM) who undertook the post-mortem examinations. A comprehensive set of post-mortem photographs of the cranial contents, including any pathological features of head injury, were also made available.

The two observing consultant forensic pathologists were asked to observe all 3 images for each of the 27 cases and to complete a questionnaire generated by Google Forms with the following questions; ‘is macroscopic IDH visible in this case?’ and if yes, ‘is the IDH focal or diffuse?’ For the purpose of this study the pathologists were requested to make a subjective assessment as to whether or not they considered IDH to be diffuse or focal. Although assessment was based on the pathologist’s experience with the use of this terminology, both agreed that they considered focal to describe small, discrete areas if IDH, whilst diffuse was used to define a large, confluent area of IDH. As part of the questionnaire, the pathologists were also asked to detail any other observations that they considered relevant to the detection of IDH.

### Statistical analysis

Inter-observer agreement between the two consultant forensic pathologists for the macroscopic detection of IDH was calculated using Cohen’s kappa coefficient. Cohen’s kappa coefficient allows inter-observer reliability testing, producing a kappa value representative of the level of agreement and reliability of data between observers, whilst excluding any agreement that may occur through chance [7]. A kappa value can range from −1 to +1 and can be interpreted as follows: ≤ 0 indicates no agreement, 0.01–0.20 indicates no to slight agreement, 0.21–0.40 indicates fair levels of agreement, 0.41–0.60 indicates moderate agreement, 0.61–0.80 substantial agreement, and 0.81–1.00 indicates almost perfect agreement [8].

Cohen’s kappa coefficient was also used to measure the level of agreement between the observing pathologists for their assessment of the extent of IDH (either focal or diffuse). Determining the level of agreement, for the extent of IDH, relies on both pathologists making a positive initial agreement on the presence of IDH. For this reason, only cases where both pathologists agreed on the presence of IDH could be analyzed for the extent of the hemorrhage.

## Results

### Occurrence of macroscopic IDH

Of the 27 cases, Pathologist A identified 12 (44%) cases with macroscopic IDH and 15 (56%) cases without. Pathologist B identified 13 (48%) cases with and 11 (41%) cases without IDH. Pathologist B was unable to determine the presence/absence of IDH in 3 (11%) cases (Table 1) due to; SDH obscuring a complete view of the tentorium and falx (case 15), a poorly focused image (case 26) and a fold within the falx which made the distinction between IDH and SDH challenging (case 27). These 3 cases were therefore excluded from statistical analysis to determine inter-observer agreement.

Analysis of the remaining 24 cases showed a kappa coefficient value of 0.669 (*p* = 0.001), indicating a substantial level of agreement for the presence of IDH between the pathologists.

### Extent of IDH: focal or diffuse

Of the 27 cases, Pathologist A reported a total of 5 (19%) cases with diffuse IDH and 7 (26%) cases with focal IDH. Pathologist B reported a total of 3 (11%) cases with diffuse IDH, and 10 (37%) cases with focal IDH (Table 1). The two observing pathologists agreed on the macroscopic presence of IDH in 10 cases. Of these 10 cases, Pathologist A reported 5 cases as focal and 5 cases as diffuse. Pathologist B reported 7 cases as focal and 3 as diffuse. This resulted in a kappa coefficient value of 0.6 (*p* = 0.038), indicating a moderate level of agreement.

### Relationship between IDH and SDH

**Fig. 1:**
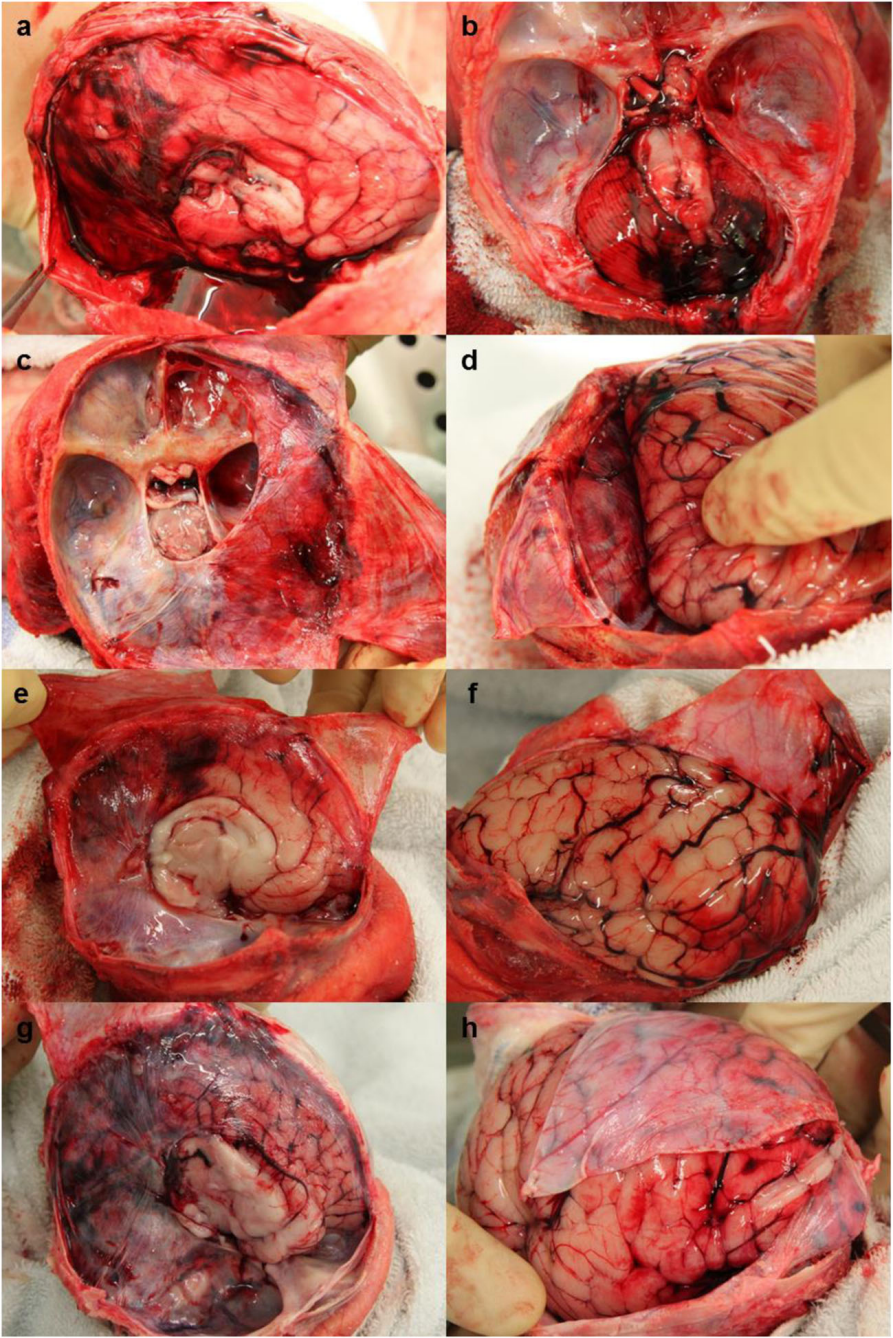
Cases with both SDH and agreed IDH. **a-b** Case 2, a 1 day old male with diffuse IDH as well as thin films of SDH over the surface of the cerebellum and within the posterior fossa and thin smears of SDH underneath the cerebrum and left occipital convexity. **c-d** Case 6, a 3 day old female with diffuse IDH and very thin smears of SDH on the parasagittal parietal dura and posterior interhemispheric fissure/occipital lobes. **e-f** Case 8, a 12 day old female with focal IDH and a very thin film of SDH over occipital and parietal lobes, within the interhemispheric fissure and under the temporal lobe. **g-h** Case 10, a 4 week old infant with diffuse IDH and a thin smear of SDH over the left occipital and parietal lobes and cerebellum

Of the 27 cases, 8 had SDH identified from the autopsy reports and photographs. Of these 8 cases, 4 also had agreed IDH, (total cases with agreed IDH was 10) (Table 1) (Fig. 1). In 3 of these 4 cases the SDH found at autopsy was described as a trivial amount of blood, presenting as a thin smear, and unlikely to be related to the cause of death. In the case of a 1 day old birth-related head injury (case 2) with diffuse IDH, a shearing tear was noted in the right side of the tentorium, as well as thin films of SDH over the surface of the cerebellum and within the posterior fossa and thin smears of SDH underneath the cerebrum and left occipital convexity.

**Fig. 2:**
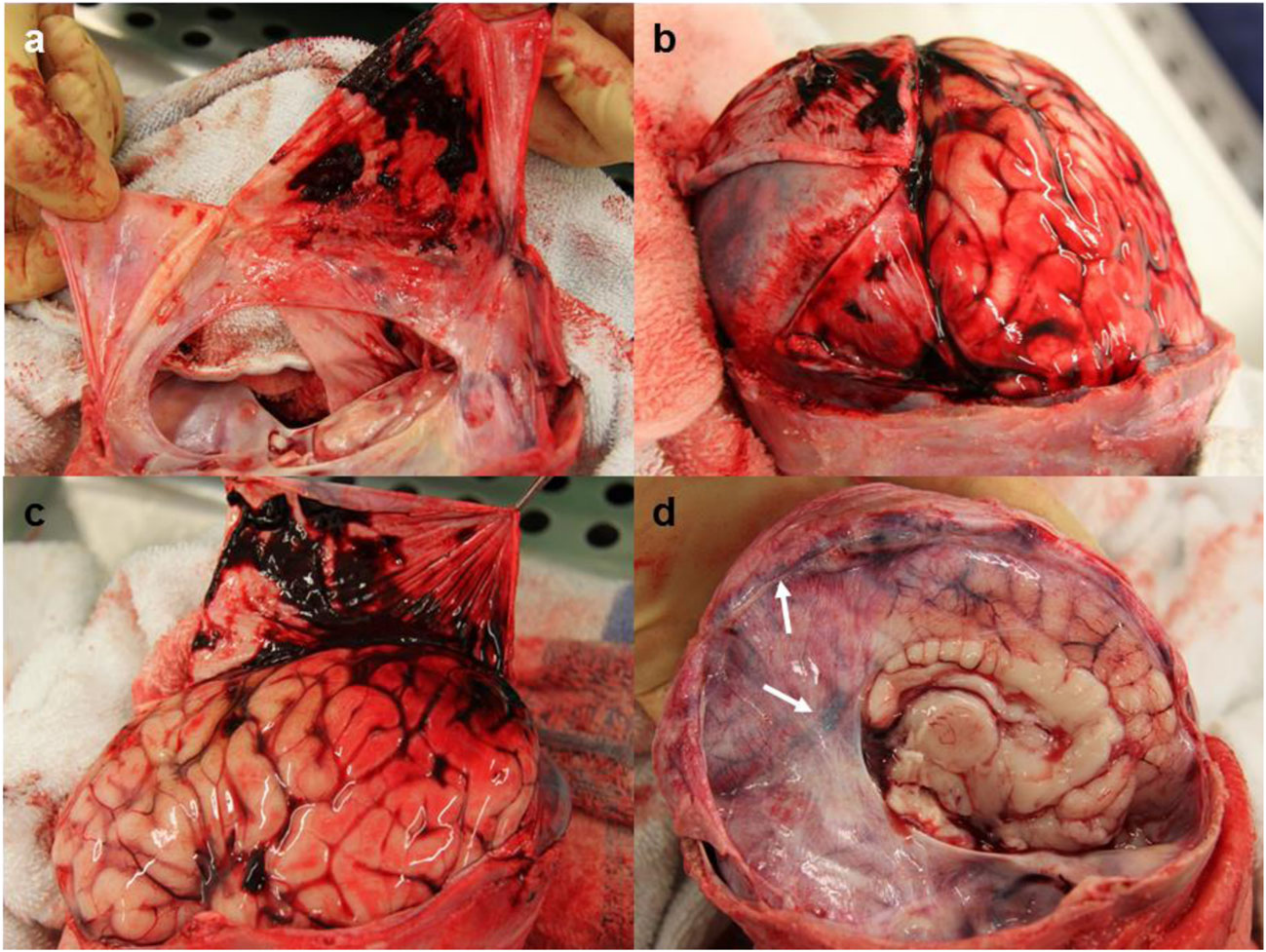
Masking SDH and blood within the sinuses. **a-c** Case 15, a 9 week old male with suspected AHT. Bilateral thin film SDH can be seen to mask certain areas of the dura, making detection of IDH challenging. **d** Case 9, a 24 day old male, blood can be seen within both the straight and superior sagittal sinuses (arrows)

In the case of a 9 week old infant (case 15) with suspected AHT the SDH was more extensive than the SDHs seen in the other cases in the series. The bleeding was distributed over the convexities, including all lobes of the brain, and within the interhemispheric fissure as patchy thin films. There was also a thin smear of SDH over the surface of the cerebellum. In this case, Pathologist A described focal IDH but Pathologist B was unable to determine the presence or absence of IDH due to masking of the dura by SDH (Fig. 2a-c).

### Relationship between IDH and age

Grouping the cases by age (Table 1) it became apparent that the majority of IDH occurred within the neonatal age group (7/9 neonatal cases (77.8%) were identified as having IDH by both pathologists, 5 of which were ≤ 3 days old). The remaining 3 cases of IDH identified by both pathologists, were amongst the infant age group, (3/14 cases (21.4%)). Of these 3 infants just one (a 4 week old male (case 10) with a cause of death of sudden unexplained death in infancy (SUDI), with uncertain sleeping arrangement) was found to have SDH at autopsy (described as a trivial thin smear over the left occipital/parietal lobe and cerebellum) (Table 1 & Fig. 1g-h). Within the young children age group there was limited inter-observer agreement for the presence of IDH in the 4 cases. Both pathologists agreed that there was no IDH in one case. In two of the cases, described earlier (case 26 and 27), Pathologist B was unable to interpret the images, so there was no agreement. Pathologist A reported no IDH for these two cases. The final case within this age group was described by Pathologist A as no IDH present, whilst Pathologist B recorded that there was focal macroscopic IDH.

### Pathologist comments on the identification of IDH

It was noted by both observing pathologists on two separate cases that the complete assessment of IDH within the falx and tentorium was challenging due to masking by SDH (Fig. 2a-c). The pathologists also frequently commented on the presence of IDH adjacent to the dural venous sinuses, in particular the superior sagittal sinus and the straight sinus (Fig. 2d).

## Discussion

The occurrence of IDH has previously been reported from autopsy photographs, post-mortem reports and histological examination of the falx and tentorium in a retrospective study [2]. In this current study, a substantial level of agreement was calculated between the two experienced observing forensic pathologists, using the same images and viewing conditions and with similar levels of experience and training. Whilst this kappa value (0.669) is a good level of agreement it is important to bear in mind that any agreement less than perfect (1.0) is a measure not only of agreement, but also the reverse, disagreement amongst the observers [8]. Therefore, the value in this study of less than 1.0 demonstrates the occurrence of cases in which the observers did not agree on the presence/absence of IDH. It has been suggested that when used for health research questions, judgements about what level of kappa should be classed as acceptable need to be made. The author of one paper has suggested an alternative interpretation of kappa in which 0.60–0.79 shows moderate agreement and 0.80–0.90 shows a strong agreement [8].

The method described in our previous publication [6] of removing each hemisphere separately provides the opportunity to observe and photographically document the falx and tentorium, in situ, including the presence of both macroscopically visible SDH and IDH. Providing access to clinical data and a previous pathologist’s interpretation of autopsy findings may influence analysis of autopsy images, and for this reason Pathologist A and B were blinded to all case histories.

Our study is congruent with the findings of previous papers [1, 3] in which IDH is more commonly seen in the younger age groups, with 7/10 of our cases with agreed IDH occurring in the neonatal group, 5 of which were ≤ 3 days old. This demonstration of IDH in this period, not long after birth, may indicate a susceptibly for IDH in this age group due to the birth, which in itself is not an atraumatic process (including potential molding of the bones due to compression) and may sometimes also require intervention such as forceps or ventouse delivery.

In an earlier combined macroscopic and histological study of dural samples taken from 55 fetuses and neonates with obvious macroscopic IDH, it was recorded that the majority of IDHs presented as diffuse in both the falx and tentorium [3]. This study also described a thin film SDH over the convexities in 16/25 (64%) fetuses and 20/30 (66.7%) neonates. Diffuse intra-falcine hemorrhage was recorded for all fetal cases with SDH and in 17/19 (89.5%) neonates in which SDH was documented (the 20th neonatal case with SDH did not have the dura examined for IDH).

A further study by the same group detailing an audit of post-mortem reports (including histology of dura) and photographs from two NHS hospitals [2] reported 55 cases of fetal SDH and 72 cases of infant and child SDH, all of which had IDH. One can deduce from the published results section and tables that all IDH were classified as ‘macroscopic’ when associated with SDH. Investigation of the 27 cases presented in our study, showed that there were 8 cases of SDH at autopsy. Of these 8, Pathologist A and B agreed on the presence of diffuse IDH in 3 cases, focal IDH in 1 case and no IDH in 3 cases. In the remaining case Pathologist A observed focal IDH and Pathologist B was unable to make an observation from the photographs. Therefore, our data does not support a direct and consistent correlation between the macroscopic presence and extent of IDH and SDH. We recognize that our small study cohort limits statistical analysis and that future investigation of a larger group would be necessary to confirm the results presented in this paper.

Accurate and consistent reporting of the macroscopic presence of IDH from autopsy photographs can be compromised due to potential SDH obscuring sections of the dura in some cases and also the differentiation between very small amounts smears of blood occurring within the dura itself or on the surface of the membrane (and in fact SDH). This may be a particular problem when viewing the falx after an initial post-mortem right hemispherectomy as SDH behind the dura, within the inter-hemispheric fissure, could potentially be mistaken for IDH. The assessment of IDH is further complicated by the presence of the blood-filled sinuses within the dura which could potentially be mistaken for IDH. For these reasons, we instructed pathologists to view both right and left aspects of the falx (after removal of both hemispheres) to make a more informed assessment. When intradural hemorrhage was present, it often appeared close to the dural venous sinuses, particularly the straight and superior sagittal sinuses (Fig. 2d). Histological examination of the tissue would be necessary to provide evidence that the blood was within the dura and not in the sinuses or one of the lesser documented smaller venous sinuses (sometimes referred to as meningeal veins or lakes) [9–11]. We did not take samples of dura for histological examination in all of our cases as this is not our routine autopsy practice when there is no macroscopically visible SDH, dural tear, or extensive IDH. However, histological examination of apparent macroscopic IDH could be considered in prospective research cases to confirm the presence and exact location of bleeding, in which the appropriate consent and ethical approvals could be obtained.

In summary, our findings in this study have highlighted a substantial level of agreement for the retrospective interpretation of post-mortem photographic images for the purpose of detection of macroscopic IDH. We have also identified a much lower frequency of macroscopically identifiable IDH occurring alongside SDH than that which has previously been described in studies which also used histology in their assessment [2, 3].

## Key points

1. There was a substantial level of inter-observer agreement between forensic pathologists when assessing autopsy photographs for the presence of macroscopic intradural hemorrhage.
2. The accurate and consistent interpretation of IDH from autopsy images can be complicated by potential artefacts such as SDH, or by blood within the dural venous sinuses.
3. The majority of cases of macroscopic IDH were seen in the early neonatal age group.
4. The majority of IDHs were focal in distribution.
5. We report a much lower frequency of macroscopic IDH occurring alongside SDH than previous studies which also used histological assessment.
